# Association of Leptin with Total and Free Testosterone: Results from the National Health and Nutrition Examination Surveys

**DOI:** 10.1089/andro.2020.0007

**Published:** 2020-10-26

**Authors:** Thiago Fernandes Negris Lima, Sirpi Nackeeran, Evgeniya Rakitina, Gustavo Fernandes Negris Lima, Himanshu Arora, Atil Y. Kargi, Ranjith Ramasamy

**Affiliations:** 1 Department of Urology, University of Miami Miller School of Medicine, Miami, Florida, USA; 2 Mechanical Engineering, Federal University of Espirito Santo (UFES), Vitoria, Brazil; 3 The Interdisciplinary Stem Cell Institute, Miller School of Medicine, University of Miami, Miami, Florida, USA; 4 Department of Human Genetics, John P Hussman Institute for Human Genomics, Miller School of Medicine, University of Miami, Miami, Florida, USA; 5 Division of Endocrinology, Department of Medicine, University of Miami Miller School of Medicine, Miami, Florida, USA

**Keywords:** leptin, testosterone, NHANES, obesity, free testosterone

## Abstract

**Introduction:**

Obese men can have testosterone deficiency (TD) but the etiology is uncertain. Leptin is a 16-kDa protein produced primarily by adipose tissue and, therefore, is positively associated with the amount of body fat and can affect testosterone (T) production. We hypothesized that increased leptin can be independently associated with low T.

**Materials and Methods:**

We performed a cross-sectional analysis of men from National Health and Nutrition Examination III database to evaluate the association of leptin with serum T and calculated free testosterone (cFT). Linear regression was performed with leptin, age, waist circumference, hypertension, and diabetes as independent variables predicting cFT/T. Multiple linear regression was used to determine predictors for cFT and T using variables previously significant in the univariate analysis.

**Results:**

A total of 1193 men were analyzed. As expected, older and obese men were associated with having lower T. Interestingly, increasing leptin levels were an independent predictor of decreasing T and cFT on multivariable analysis. Increasing 1ng/mL in leptin resulted in a decrease of 5.13 and 0.11 ng/dL of T and cFT, respectively (*p* < 0.05). Also, every additional year of life led to a T and cFT reduction of 2.87 and 0.13 ng/dL, respectively, and increasing 1 cm in waist circumference corresponded to decrease of 4ng/dL in T (*p* < 0.05).

**Conclusions:**

We concluded that increasing leptin, age, and waist circumference were associated with decreasing of T and cFT. Elevated leptin levels could be one of the potential etiologies of TD.

## Introduction

Worldwide obesity has tripled since 1975. According to the World Health Organization, in 2016, 1.9 billion (39%) of adults were overweight. Of these >650 million (13%) were obese.^1^ Recent clinical evidence suggests that obesity is one of the most important risk factors for secondary hypogonadism in men.^2^

Many mechanisms have been suggested to cause low total testosterone (T) in male obese patients including lower sex hormone binding globulin (SHBG) levels due to a suppression of hepatic SHBG synthesis by elevated concentrations of insulin (*in vitro* study with Hep G2 cells),^3^ reduced SHBG caused by the elevated concentration of lipid in the liver, and by high content of proinflammatory cytokines,^4^ elevated peripheral conversion of T to estradiol (E_2_), and its negative feedback to the hypothalamic–pituitary–gonadal (HPG) axis,^5^ and direct and indirect effects of leptin on the HPG axis.^6^

Leptin is a 167 amino acid peptide, product of the *ob* gene, mainly produced in white adipose tissue. It regulates energy balance by optimizing energy expenditure, inhibiting food intake, and it is strongly correlated (positive) with the amount of body fat.^7^ Thus, weight loss is associated with reduction of leptin levels.^8^ Leptin has not only been associated with regulation of body fat stores but also has clear regulatory functions within the HPG axis.^9^

In a state of decreased energy supply, a decreased leptin level has been found to be a key mediator of neuroendocrine abnormalities seen in hypogonadotropic hypogonadism.^6,10^ In contrast, high-fat diet-induced obesity resulted in elevation of leptin and downregulation of kisspeptins and gonadotropin-releasing hormone (GnRH) secretion in the hypothalamus of mice and humans,^11^ possibly contributing to male hypogonadotropic hypogonadism.^12^ Interestingly, Leydig cells have leptin receptors as well.^13^ It has been suggested that high serum levels of leptin could actively impair 17,20 lyase steps of the testicular steroidogenic pathway, causing decreased intratesticular T production.^13^

Several studies suggest that the regulation of testicular steroidogenesis is influenced by leptin among other hormones.^6,12–14^ The gap in knowledge is to understand the underlying etiology for testosterone deficiency (TD) in obese men. We hypothesized that serum leptin would be inversely associated with T and free testosterone (FT) levels. We examined the National Health and Nutrition Examination Survey (NHANES), assessing demographic characteristics, serum leptin, and T. We have also calculated FT using total T, SHBG, and albumin.

## Materials and Methods

### Data source and study population

This study is a cross-sectional analysis of 1193 men of ages 20–90 years based on the NHANES III database survey from 1988 to 1991 and available online.^15^ The NHANES is a collection of studies designed by the Center for Disease Control and Prevention (CDC) to determine the dietary status and overall health of individuals within the United States.^16^ Only the 1988–1991 data cycle was included, because other data cycles were either missing in T or leptin data. Baseline characteristics of age, race, waist circumference, diabetes, hypertension, and smoking status, as well as leptin and T levels, were assessed. Subjects were evaluated according to their waist circumference since it was previously reported to be a better predictor of obesity-related health risk in the NHANES III population.^17,18^

The NHANES collects its data through a complex multistage probability sampling design with testing conducted at several distinct sites. Owing to this methodology, certain subgroups are overrepresented in the NHANES sample. To account for this, the NHANES assigns sample weight, variance, and cluster variables to adjust the sample population to the United States census. All analyses for this study were conducted utilizing these sample weights, variances, and clusters as recommended by the NHANES guidelines.

### Ethics statement

Per the National Center for Health Statistics Ethics Review Board, all participants submitted a written consent to be enrolled in this study (https://www.cdc.gov/nchs/nhanes/irba98.htm).

### Covariables and primary outcome

Our primary outcome was to determine the association between serum leptin, calculated free testosterone (cFT), and T levels after excluding possible confounders, such as age, history of diabetes, history of hypertension, and waist circumference. Since cigarette smoking was not associated with low T, we excluded it from the analysis.^19^ A competitive electrochemiluminescence immunoassay on the 2010 Elecsys autoanalyzer (Roche Diagnostics, Indianapolis, IN) was used to quantify serum T, E_2_, and SHBG concentrations, which were archived in the NHANES database.

T results were obtained in ng/mL and converted to ng/dL, since this is the unit used most commonly in clinical practice.^20^ FT and free estradiol (FE) were also calculated from SHBG, albumin, and T using the method of Vermeulen et al.,^21^ relying on the assumption that the concentration of these fractions is determined mainly by the interaction of albumin, SHBG with total T or total E_2_.^22^ Serum leptin concentrations were measured by radioimmunoassay after storage at −70°C for 8 years and after one freeze–thaw cycle on average.^23^ As a secondary outcome, we assessed the association between waist circumference and leptin levels in a multiple regression analysis, to validate our findings with previous studies.^5,7,9^

### Statistical analysis

Statistical analysis was performed using SAS® version 9.4 (SAS Institute, Inc., Cary, NC) and GraphPad Prism version 8 (GraphPad Software, Inc.). All data analyzed were weighted using the appropriate strata and cluster per CDC recommendations. Measures of central tendencies and variability were reported as mean and standard deviation, respectively. *t*-Test was used to compare continuous variables and chi-squared test was used to compare categorical variables.

We performed univariate linear regression analysis using leptin, E_2_, age, waist circumference, hypertension, and diabetes to determine which variables would have an effect on cFT and T levels. Subsequently, a multiple linear regression was used to determine the best predictors for cFT and T, controlling for all confounding variables that were significant in the univariate analysis. Participants were included regardless of smoking status. We also performed a multiple linear regression analysis including age, T, waist circumference, diabetes, and hypertension to predict leptin levels.

To avoid multicollinearity, we confirmed that none of the analysis variables had a high degree of correlation through calculation of tolerance for each independent variable. In addition, we calculated the influence of data points through the Cook’s distance of each case and the Durbin-Watson test was performed to assess autocorrelation of the residuals in each model. The significance level was set at *p* < 0.05.

## Results

A total of 1193 men >20 years of age had baseline characteristics, leptin, and T levels for the analysis. The clinical and demographic information is given in Table 1. As expected from a population sample, most men had normal T, 542.6 ng/dL (95% confidence interval 524.9–560.35). The prevalence of men with T < 300 ng/dL was 8.9% (106 men). In addition, the prevalence of diabetes and hypertension in this population was 3% and 19%, respectively.

In the simple linear regression analysis, most variables (leptin, age, waist circumference, hypertension, and diabetes) were negatively associated with variation in T levels (*p* < 0.05), whereas E_2_ was only positively associated with T levels (*R*^2^ = 0.13, *p* = 0.0011). When evaluating predictors for cFT, we observed a similar pattern, where leptin, age, waist circumference, hypertension, and diabetes negatively predicted cFT levels (*p* < 0.05); E_2_ positively predicted cFT levels (*R*^2^ = 0.16, *p* = 0.0006). FE was not associated with variation of T or cFT levels (*p* > 0.05, *R*^2^ = 0.00) (Table 2).

Including only the significant independent variables from the simple linear analyses, we proceeded with a multiple linear regression analysis to predict T and cFT levels. The analysis showed that increasing leptin, age, and waist circumference negatively predicted T levels (*p* < 0.05), and as expected, E_2_ was a positive predictor (*p* = 0.0024). A similar pattern held true for the predictors of cFT levels, where leptin levels (*p* = 0.0004) and age (*p* < 0.001) showed negative association with cFT, whereas E_2_ showed a positive association (*p* = 0.0014). Our model predicted 38% and 47% of the variability of T and cFT levels, respectively (T *R*^2^ = 0.38, cFT *R*^2^ = 0.47). According to our findings, an additional 1 ng/mL in leptin levels resulted in a reduction of 5.13 and 0.11 ng/dL of T and cFT, respectively (*p* < 0.05). Also, we observed a reduction of 2.87 ng/dL in T and 0.13ng/dL in cFT for every additional year of life (*p* < 0.05). Moreover, we perceived a reduction of 4 ng/dL in T levels (*p* < 0.05) for every additional 1 cm in the waist circumference (Table 3). Older and obese men were associated with lower levels of T. Interestingly, men with higher leptin levels had lower T, independent of waist circumference and age.

In the multivariate linear model to predict leptin level, waist circumference and age were the only significant variables. For every additional 1 cm of waist circumference, we observed an increase of 0.27 ng/mL (*p* < 0.0001). Conversely, the addition of 1 year of age led to a decrease of 0.04 ng/mL in leptin levels (*p* < 0.05). This model predicted 50% variability of leptin levels (*R*^2^ = 0.50) (Table 4).

## Discussion

Besides its proven relationship with body fat,^7^ leptin, in high levels, is believed to inhibit the testicular steroidogenic pathway^24^ and downregulate GnRH production and secretion,^11,25^ causing low T levels in men. This finding could justify the higher incidence of low T in the obese population when compared with the nonobese. The purpose of this cross-sectional analysis was to evaluate the negative association between serum leptin, FT, and serum T in men. Variables, such as age, leptin, and waist circumference negatively impacted T levels, giving support to previous studies that suggested that metabolic syndrome and insulin resistance were related to hypogonadism in men.^26,27^ Curiously, only leptin and age were negatively associated with cFT levels.

Previous studies have demonstrated that the regulation of leptin and T levels seems to be bidirectional.^28,29^ Our population level study supports previous findings in smaller sample size studies that establish negative associations between leptin and serum T^28^ and a positive association between leptin and obesity.^7,8^ The presence of a significant association between leptin and T independent of waist circumference indicates that elevated leptin levels could lead to TD regardless of body composition. This relationship is further supported by prior studies that revealed leptin’s effect on Leydig cell steroidogenesis in murine models.^30^ It is likely that the high leptin levels in obese patients play a role independent of the other factors involved in obesity on comorbid hypoandrogenism.^31^ In contrast, another study suggested that low T could impact leptin levels.^32^ In this study, castrated rats presented with the highest levels of leptin when compared with controls and those receiving exogenous T.^33^ Since the majority of men in this study had normal serum T, low T was not a predictor of elevated leptin levels (Table 4).

Many studies have associated leptin impact with T production associated with body mass index (BMI). Waist circumference was considered to be a more reliable predictor of obesity-related health risk for the NHANES population, based on Janssen et al.^18^ study assessing waist circumference as a predictor of obesity-related health risk, besides its good association with central obesity and metabolic syndrome.^18^ In addition, in contrast to previous findings,^5,27^ our study showed a positive association between E_2_ and T and cFT levels. Since estrogen production in men depends on testicular or peripheral aromatization of androgens,^33^ we expected to see a positive association between T and E_2_.^34^

Although obesity is classically a cause of secondary hypogonadism,^2,5^ the frequently elevated concentration of leptin in these individuals appears to directly affect testicular function. To analyze the direct effect of leptin on the testicular function, Isidori et al.^35^ examined the *in vivo* relationship between leptin levels and sex hormones. In this study, men were subjected to human chorionic gonadotropin (hCG) stimulation (5000 IU) and blood work was monitored for 4 consecutive days. The authors observed that androgen levels were inversely correlated with leptin levels after hCG stimulation. Also, the decreased androgen response to hCG stimulation in obese men was not related to increased aromatase activity and consequent peripheral conversion of T to E2, since no significant changes in E2/T ratio between obese men and lean men were noted. These results suggest that obesity-related TD may not only be related to secondary hypogonadism, but also caused by a deficient testicular response to adequate stimulus. Therefore, leptin appears to have an important role in the regulation of the HPG axis and in the pathogenesis of TD in obese men.

The negative correlation between aging and leptin levels has been previously described.^36^ Leptin levels appear to be reduced in >50% in men older than 60 years.^37^ Although in our population, leptin was also negatively associated with age, we observed a less accentuated association, with variation of 0.04 ng/mL for every year.

Strengths of this study include the very large sample size and the broad range of waist circumference and age, which allows for robust statistical inferences regarding the factors influencing SHBG concentrations. Given the subject pool of NHANES III and its validated weighting methods, this study possibly provided a representative sample of the American population.

Limitations of this study include its retrospective and cross-sectional design. Although our findings of predictors of T and cFT levels were statistically significant, they yielded predictions that should not be used in clinical practice independently. We understand that these findings can be justified by a multifactorial etiology of hypogonadism that includes genetic conditions, anatomic abnormalities, infection, tumor, medication, comorbidity, and injury.^38^ Furthermore, the lack of data regarding symptoms of hypogonadism in the NHANES database prevented us from analyzing a diagnosis of hypogonadism as an outcome in this study. In addition, our assessment of FT was calculated based on the T and SHBG concentrations as measured with an immunoassay, rather than the gold standard of equilibrium dialysis for the measurement of FT. In contrast, the cFT used in our analyses has been shown to correlate highly with FT measured by equilibrium dialysis and represents a practical measure that is used clinically.^21^

Another limitation was that we did not have access to the list of symptoms, medications, and medical history for each patient. Also, key analysis variables were only available for the NHANES data set from 1988 to 1991 for men aged 20 years and older, which may not reflect the exact demographic characteristics of the 2020 male population. In contrast, since obesity prevalence has only increased from the data collection period to the present time, we expect a crescent impact of leptin on T production.

Previous studies showed that FT was especially affected in the severe obese male population (BMI >35–40 kg/m^2^).^4^ Since only a small percentage of men in our study had BMI >35 kg/m^2^, we could not replicate this association in the study (Table 1). Our study was additionally limited by the lack of luteinizing hormone data in the NHANES database, which could have served to understand whether the effect of leptin is primarily at the level of the testis or secondarily at the hypothalamus/pituitary gland. Therefore, our conclusions apply only to the measurement of low T and cFT and not to the diagnosis of hypogonadism, besides having the limitation of not including all possible predictor variables.

## Conclusions

In a large sample representative of the American population, increasing serum leptin is independently associated with both decreasing total and FT levels, even when controlling for other associated factors. Leptin is one of the several factors that can explain the underlying association between obesity and TD.

## Figures and Tables

**Table 1.** Baseline Characteristics of Participants Meeting Inclusion Criteria

Variables	Mean (95% CI)
Gender^a^	
Men (%)	100 (100–100)
Women (%)	0 (0–0)
Age (years)^b^	42.1 (40.6–43.6)
Testosterone (ng/dL)^b^	542.6 (524.9–560.35)
Free testosterone (ng/dL)^b,c^	11.0 (10.6–11.4)
Leptin (ng/mL)^b^	5.8 (5.3–6.4)
Sex hormone binding globulin (nmol/L)^b^	37.7 (36.2–39.2)
Estradiol (pg/mL)^b^	37.5 (36.0–39.0)
Free estradiol (pg/mL)^b^	0.64 (0.62–0.67)
Waist circumference (cm)^b^	94.7 (93.3–96.0)
BMI categories (kg/m^2^)^a^	
<25 (%)	44.1 (40.6–47.7)
25–29.9 (%)	37.6 (33.7–41.5)
30-34.9 (%)	13.4 (10.6–16.2)
≥35 (%)	4.9 (2.8–6.9)
Diabetes^a^	
No (%)	97.0 (95.9–98.1)
Yes (%)	3.0 (1.9–4.1)
Hypertension^a^	
No (%)	81.0 (78.4–83.5)
Yes (%)	19.0 (16.5–21.6)
Race^a^	
Black (%)	9.7 (6.8–12.5)
Mexican (%)	4.8 (2.9–6.8)
White (%)	78.4 (72.0–84.8)
Other (%)	7.1 (2.7–11.5)
Testosterone <300 ng/dL^a,d^	
No (%)	91.1 (89.0–93.3)
Yes (%)	8.9 (6.7–11.0)

a Percentages adjusted to account for complex survey design and unweighted numbers.

b Mean value of continuous variables in the analysis population with values and confidence limits adjusted to account for complex survey design and unweighted numbers.

c Values calculated according to previously validated methods (Vermeulen).

d Patients included by serum T only as symptoms of clinical hypogonadism were not recorded in the NHANES database.

BMI, body mass index; CI, confidence interval; NHANES, National Health and Nutrition Examination Survey.

**Table 2.** Simple Linear Regression Investigating Predictors of Total Testosterone and Calculated Free Testosterone

Dependent variable	Total testosterone (ng/dL)						Calculated free testosterone (ng/dL)					
			95% CI						95% CI			
Independent variable	*B*	SE	Lower	Upper	*p*	*R*^2^	*B*	SE	Lower	Upper	*p*	*R*^2^
Leptin (ng/mL)	−12.59*	2.97	−18.73	−6.46	0.0003	0.13	−0.19*	0.05	−0.29	−0.09	0.0005	0.06
Estradiol (pg/mL)	5.89*	1.59	2.61	9.17	0.0011	0.13	0.14*	0.04	0.07	0.22	0.0006	0.16
Free estradiol (pg/mL)	−7.44	32.55	−74.79	59.90	0.8211	0.00	0.31	0.50	−0.74	1.35	0.5514	0.00
Age (years)	−4.36*	0.55	−5.50	−3.23	<0.0001	0.13	−0.15*	0.01	−0.17	−0.13	<0.0001	0.32
Waist circumference (cm)	−6.38*	0.51	−7.43	−5.34	<0.0001	0.20	−0.10*	0.01	−0.12	−0.07	<0.0001	0.10
Hypertension (yes vs. no)	−74.71*	22.50	−121.25	−28.17	0.0030	0.02	−1.93*	0.48	−2.92	−0.93	0.0006	0.03
Diabetes (yes vs. no)	−134.39*	18.15	−171.93	−96.85	<0.0001	0.01	−2.92	0.41	−3.78	−2.06	<0.0001	0.01

* *p* < 0.05.

SE, standard error.

**Table 3.** Multiple Linear Regression Model Predicting Total Testosterone (ng/dL) and Calculated Free Testosterone (ng/dL)

	Total testosterone					Calculated free testosterone				
			95% CI					95% CI		
Variable	*B*	SE	Lower	Upper	*p*	*B*	SE	Lower	Upper	*p*
Leptin (ng/mL)	−5.13*	2.20	−9.68	−0.59	0.0285	−0.11*	0.03	−0.17	−0.06	0.0004
Estradiol (pg/mL)	5.38*	1.58	2.12	8.64	0.0024	0.12*	0.03	0.05	0.20	0.0014
Age (years)	−2.87*	0.43	−5.61	−2.39	<0.0001	−0.13*	0.01	−0.15	−0.11	<0.0001
Waist circumference (cm)	−4.00*	0.78	−5.61	−2.39	<0.0001	−0.02	0.01	−0.05	0.00	0.0963
Diabetes (yes vs. no)	−8.40	22.59	−55.13	38.32	0.7133	0.01	0.41	−0.83	0.85	0.9794
Hypertension (yes vs. no)	10.62	21.86	−34.59	55.83	0.6316	−0.13	0.43	−1.02	0.76	0.7659

*R*^2^ T = 0.38; *R*^2^ cFT = 0.47.

* *p* < 0.05.

T, testosterone; cFT, calculated free testosterone.

**Table 4.** Multiple Linear Regression Model to Determine Associations with Leptin

			95% CI		
Variable	*B*	SE	Lower	Upper	*p*
Waist circumference (cm)	0.27*	0.03	0.21	0.33	<0.0001
Total testosterone (ng/mL)	−0.25	0.14	−0.53	0.03	0.0799
Age (years)	−0.04*	0.02	−0.07	−0.00	0.0402
Diabetes (yes vs. no)	−0.40	1.31	−3.10	2.31	0.7647
Hypertension (yes vs. no)	0.87	0.48	−0.12	1.87	0.0821

*R*^2^ = 0.50.

* *p* < 0.05.

## Abbreviations Used

BMI: body mass index
CDC: Center for Disease Control and Prevention
cFT: calculated free testosterone
CI: confidence interval
E_2_: estradiol
FE: free estradiol
FT: free testosterone
GnRH: gonadotropin-releasing hormone
hCG: human chorionic gonadotropin
HPG: hypothalamic-pituitary-gonadal
NHANES: National Health and Nutrition Examination Survey
SE: standard error
SHBG: sex hormone binding globulin
T: testosterone
TD: testosterone deficiency
